# Deep cutaneous ulcers and sinus formation in an immunocompetent adult^☆^^☆☆^

**DOI:** 10.1016/j.abd.2018.12.003

**Published:** 2019-10-24

**Authors:** Qiang Zhou, Kejian Zhu

**Affiliations:** Department of Dermatology, Sir Run Run Shaw Hospital, Zhejiang University, Zhejiang, China

**Keywords:** Cryptococcosis, Infection, Skin ulcer

## Abstract

This report describes a case of unusual deep skin ulcers with tortuous sinus tract formation in an immunocompetent woman. She was initially diagnosed with a *Staphylococcus aureus* skin infection and histopathologically diagnosed with pyoderma gangrenosum. However, culture from the deep end of ribbon gauze inserted into the subcutaneous sinus tract revealed shiny, light-yellow mucoid colonies, which were identified as *Cryptococcus neoformans var. grubii.* She was treated with fluconazole for nine months and completely healed. Cryptococcosis is an opportunistic infection caused by variants of *C. neoformans* species. Cutaneous manifestations of cryptococcosis are quite divergent, rarely occurring as deep skin ulcers with sinus formation.

A 43-year-old woman developed skin ulcers with slight pain and purulent discharge on her left posterior axillary fold for four months and left shoulder for two months. She had been healthy before, without underlying causes of immunosuppression. She had had no contact with animals and birds. Physical examination was unremarkable except for the two deep cutaneous ulcers (Fig. 1). Serological exams were all negative. Magnetic resonance imaging revealed soft tissue and muscle infection with sinus formation. A sinogram demonstrated tortuous irregular sinus tracts extending from the two cutaneous ulcers (Fig. 2). Histological analysis displayed ulceration with dense infiltration of neutrophils. The repeated routine cultures found 100% *Staphylococcus aureus*, while the patient was not responsive to antibiotics. However, culture from the deep end of ribbon gauze inserted into the sinus tract detected shiny, light-yellow mucoid colonies (Fig. 3). India ink stain showed characteristic capsulated budding yeast cells with halos (Fig. 4). The cerebrospinal fluid examination and blood culture were negative. The biochemical and genetic identification proved that the isolated pathogen was *Cryptococcus neoformans var. grubii*. *C. neoformans* is usually recovered from soil contaminated with avian excreta, especially pigeon droppings, and decaying wood, fruits, vegetables, and dust.^1^ Cryptococcosis is an opportunistic fungal infection that occurs more commonly among immunocompromised patients with acquired immune deficiency syndrome or other underlying diseases (*e.g.*, diabetes mellitus, liver cirrhosis, and malignancies), and in subjects under immunosuppressive therapy.^2^, ^3^ However, there are also reports of cryptococcosis in immunocompetent patients.^4^, ^5^ The authors herein describe a case of *C. neofromans var. grubii* infection presenting as unusual deep skin ulcers with sinus formation but free of any clinical evidence of systemic diseases. In humans, *C. neoformans* causes three types of infections: pulmonary cryptococcosis, cryptococcal meningitis, and cutaneous cryptococcosis.^2^ Though considered as a distinct clinical entity,^1^ cutaneous cryptococcosis is mostly believed to be attributed to inhalation of *Cryptococcus* spores and later hematogenous dissemination.^2^ The diagnosis of cutaneous cryptococcosis is often difficult because the skin lesions are non-specific and have various clinical manifestations, such as cellulitis, plaques, ulcerations, pustules, granulomata, abscesses, and herpetiform or molluscum contagiosum-like lesions.^6^ However, almost any type of skin lesion – including superficial skin ulcers – can be seen in disseminated cryptococcosis, deep ulcers with sinus tract formation are very rare and have not yet been reported. It was the radiographic images illustrating soft tissue infection and existence of sinus tracts that triggered the authors’ critical idea of performing a culture from the terminal portion of the ribbon gauze inserted into the deep sinus tract, which finally produced a positive result for *C. neoformans var. grubii*. The treatment for cryptococcosis depends on the anatomical site involved and immune status of the host. According to the guidelines of the Infectious Diseases Society of America,^7^ the patient was treated with fluconazole 400 mg daily for nine months, and a complete cure was observed. This case highlights that unusual deep cutaneous ulcers with sinus tract formation may sometimes be the only manifestation of disseminated cryptococcosis and should be included in the differential diagnosis of cutaneous ulcerative lesions.

**Figure 1:**
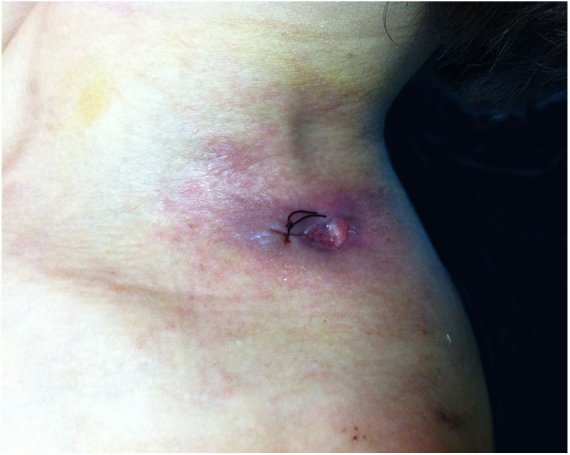
Ulcer on the left shoulder. The cutaneous ulcer on her left shoulder, after a skin biopsy.

**Figure 2:**
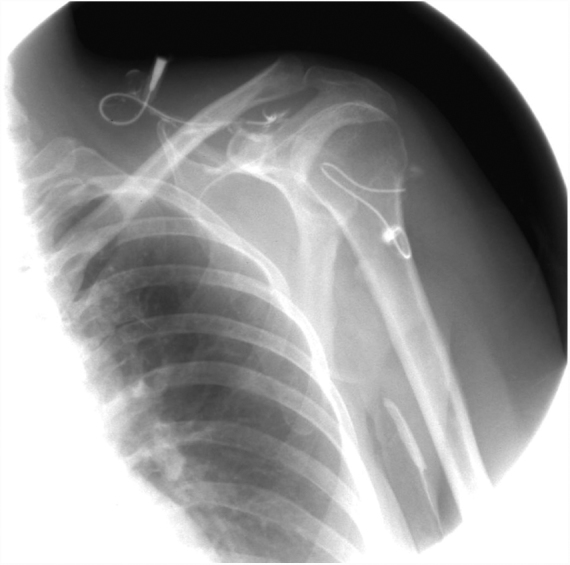
Irregular subcutaneous sinus tracts. A sinogram illustrating irregular sinus tracts extending from the cutaneous ulcers.

**Figure 3:**
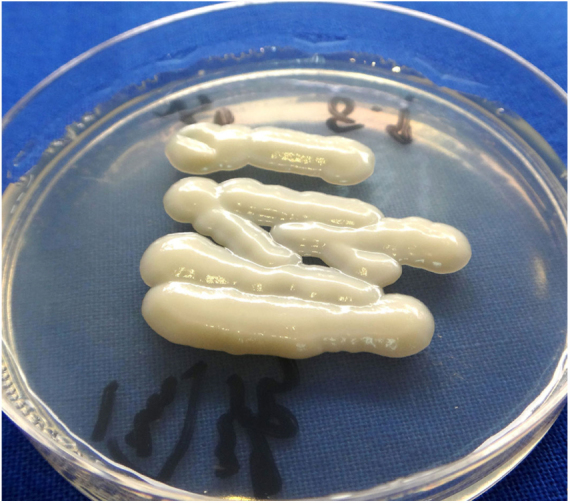
The cultured microorganism. Shiny, light-yellow mucoid colonies on Sabouraud's dextrose agar.

**Figure 4:**
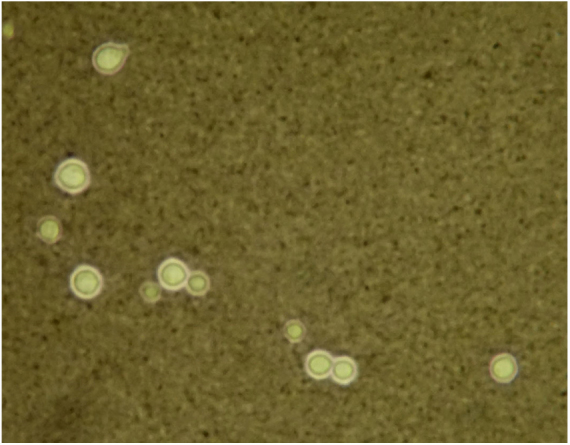
The special microorganism stain. The India ink stain showed characteristic capsulated budding yeast cells with distinct halos (x40).

## Author's contributions

Qiang Zhou: Approval of the final version of the manuscript; elaboration and writing of the manuscript; obtaining, analyzing and interpreting the data; effective participation in research orientation; intellectual participation in propaedeutic and/or therapeutic conduct of the cases studied; critical review of the literature.

Kejian Zhu: Approval of the final version of the manuscript; critical review of the manuscript.

## Conflicts of interest

None declared.

## Financial support

This work was supported by the National Natural Science Foundation of China (Grant No. 81573057).
